# Has Otitis Media Disappeared during COVID-19 Pandemic? A Fortuitus Effect of Domestic Confinement

**DOI:** 10.3390/jcm10132851

**Published:** 2021-06-27

**Authors:** Sara Torretta, Barbara Cantoni, Giuseppe Bertolozzi, Pasquale Capaccio, Gregorio Paolo Milani, Lorenzo Pignataro, Sebastiano Aleo, Paola Marchisio

**Affiliations:** 1 Fondazione IRCCS Ca’ Granda Ospedale Maggiore Policlinico, ENT and Head and Neck Surgery Unit, 20122 Milan, Italy; pasquale.capaccio@unimi.it (P.C.); lorenzo.pignataro@unimi.it (L.P.); 2 Department of Clinical Sciences and Community Health, Università degli Studi di Milano, 20122 Milan, Italy; gregorio.milani@unimi.it; 3 Fondazione IRCCS Ca’ Granda Ospedale Maggiore Policlinico, Pediatric Emergency Department, 20122 Milan, Italy; barbara.cantoni@policlinico.mi.it (B.C.); giuseppe.bortolozzi@policlinico.mi.it (G.B.); 4 Department of Biomedical Surgical Dental Science, Università degli Studi di Milano, 20122 Milan, Italy; 5 Fondazione IRCCS Ca’ Granda Ospedale Maggiore Policlinico, Pediatric Highly Intensive Care Unit, 20122 Milan, Italy; sebastiano.aleo@policlinico.mi.it (S.A.); paola.marchisio@unimi.it (P.M.); 6 Department of Pathophysiology and Transplantation, Università degli Studi di Milano, 20122 Milan, Italy

**Keywords:** otitis media, children, emergency, COVID-19, infection

## Abstract

Background: To measure patient flow at our Pediatric Emergency Department (PED) during the Italian lockdown, with particular care in terms of otolaryngological (ENT)-related diagnoses. Methods: A retrospective evaluation of electronic charts of children admitted to our PED in the City Center of Milan (Italy) for any disease. The outcome was to compare distribution of diagnoses performed at our PED during 21 February–3 May 2019 (period 1) to 21 February–3 May 2020 (period 2). Results: A total of 4538 children were evaluated during period 1 compared to 1310 during period 2. A statistically significant overall effect on diagnosis between the study periods was attested (*p*-value < 0.001; pseudo R2 = 0.010), ENT-related diagnoses being more frequently documented in period 1 (80.4% vs. 19.5%; *p*-value < 0.001), as well as those related to middle ear infections (92.8% vs. 7.2%; *p*-value < 0.001). Non-complicated acute otitis media more frequently occurred in period 1 (92.0% vs. 8.0%; *p*-value < 0.001); no significant difference in the number of complicated middle ear infections occurred (95.8% vs. 4.2%). Conclusions: The exceptional circumstances of the Italian lockdown resulted in a significant decrease in patients’ attendance to our PED, especially when considering diagnoses related to any ENT disorder, middle ear disease, and non-complicated middle ear infection.

## 1. Introduction

The SARS-Cov-2 (COVID-19) pandemic lockdown from March to May 2020 brought a deep disruption in social habits and daily activities, as well as a reduction in all medical activities other than those strictly related to emergency or management of COVID-19 patients.

Milan, one of the hardest-hit cities during the Italian epidemic, was under lockdown between 9 March and 17 May, during which all elective medical activities were halted to both address the emergent pandemic and avoid unnecessary gatherings [1].

The constraints imposed during this period totally altered people’s way of living and consisted not only in outdoor social distancing but also in domiciliary confinement, drastically cutting any outdoor activity and contact with people other than the ones strictly belonging to the domestic setting. The reduction of social interactions, child day-care attendance, and air pollution all probably help explain the positive impact on upper airway infections in children. As a fact, we recently documented a significant clinical and subjective improvement in otitis media complaints in otitis-prone children followed at our tertiary outpatient clinic for upper respiratory tract infections who had had a follow-up visit scheduled during the lockdown and were instead reached by means of telemedicine assessment via telephone calls [2]. A sharp decrease in the diagnoses of common infectious diseases among children, including otitis media, during the COVID-19 pandemic, was also reported by Hauton et al. [3].

This observation prompted us to measure patient flow at our Pediatric Emergency Department (PED) during lockdown, with particular care in terms of diagnoses related to any otolaryngological (ENT) disease (including otitis media complaints) and to compare these numbers with those observed the year before during the same period.

## 2. Materials and Methods

On 4 May 2020, at the end of the Italian lockdown for the COVID-19 pandemic, we started retrospectively assessing the electronic charts of children who had accessed the PED (De Marchi building) of our hospital (Fondazione IRCCS Ca’ Granda Ospedale Maggiore Policlinico, placed in the City Center of Milan, Lombardy) from the beginning of the COVID-19 Italian epidemic (21 February–3 May 2020; i.e., period 2).

Our hospital has three different Emergency Departments: one for the general adult population, one reserved for the management of obstetric and gynecological emergencies, and the PED for patients under 18 years. Our PED, which is a few minutes’ walk from the Duomo square, guarantees 24-hour urgent and emergency healthcare to pediatric patients in the densely populated area of Milan city (about 199,670 children aged between 0–18 years, with the 0–14 age bracket corresponding to 13.1% of the total population in Milan). In addition, our PED is the only one in Milan city and its hinterland (about 486,770 children as a whole) having a 24-hour ENT guard duty; therefore, even if other PEDs are present in the same area, in the case of pediatric ENT urgency, our hospital is the most likely alternative, or the patient is addressed here from elsewhere.

Demographic data, as well as primary discharge diagnosis based on International Classification of Disease-9 (ICD-9) codes [4], of all the children visiting our PED were recorded; gathered data were then compared with the information relative to the corresponding period in 2019 (21 February–3 May 2019; i.e., period 1).

Since the detection on 21 February of the first Italian cluster in Codogno (a small city near Lodi, placed in Lombardia), several and progressively restrictive measures were adopted in our region and maintained during all the study period (period 2). In particular, some cities in the northern Italy (mainly belonging to the Lombardia and Veneto regions) were placed under quarantine on 21 February, and on 25 February restrictive measures were extended to seven regions in northern Italy beside Lombardia. Restrictive measures consisted of human isolation within the red zones, interruption of any public manifestation, commercial and working activity not strictly considered as public utility, sporting and recreational activity, and school. Human assemblages were prohibited, and people were encouraged to wear personal protective equipment. On 1 March different restrictive measures were applied nationwide on the basis of distinctive geographic areas, and on 4 March the Italian government announced the application of unvarying measures nationwide, including suspension of teaching activities in schools of all grades and universities. On 7 March a new national administrative order abolished the red zones and prohibited all movement to and from the territories subject to restriction (i.e., Lombardia and the other 14 provinces in central and northern Italy, affecting about 16 million people), as well as within the territories themselves. These measures were extended to all the nation on 9 March; on 21 March the closure of all activities not strictly necessary for the production chain was decreed, and on 22 March a new ordinance prohibited the transfer by any public or private means of transport to different municipalities, except for proven work needs, absolute urgency, or for reasons of health. These measures were mandated until the end of the lockdown, which took place on 3 May.

The statistical analysis was mainly designed to detect any possible difference in the number and characteristics of the patients presenting to the PED during the abovementioned periods, with particular focus on diagnoses related to middle ear infections (groups 1–3), and more generally on diagnoses related to any other ENT complaint (groups 4–6) (Table 1). Diagnoses other than the ones related to the ENT are labeled in group 7.

The results are given as absolute numbers and percentages or arithmetical mean values ± standard deviation. Comparison between periods 1 and 2 in terms of demographic variables was assessed by contingency table analysis (Fisher’s exact test) for dichotomous variables and Student’s *t* test for continuous variables (after using the Shapiro–Wilk W test for normal data). The relationship between a specific diagnosis and the corresponding period was calculated employing Fisher’s exact test and factorial logistic regression analysis.

The data were analyzed using STATA 10.0 software (StataCorp, College Station, TX, USA); a *p*-value of <0.05 was considered statistically significant.

The protocol belongs to the project “Studio multicentrico italiano sulle caratteristiche cliniche e immunologiche dell’infezione da virus SARS-Cov-2 in età pediatrica” promoted by SITIP Società di Infettivologia Pediatrica e SIP Società italiana di Pediatria (2082_OPBG_2020), and it was approved by the Ethics Committee of Fondazione IRCCS Ca’ Granda Ospedale Maggiore Policlinico. This clinical observation was conducted in accordance with the principles of good clinical practice.

## 3. Results

**Figure 1:**
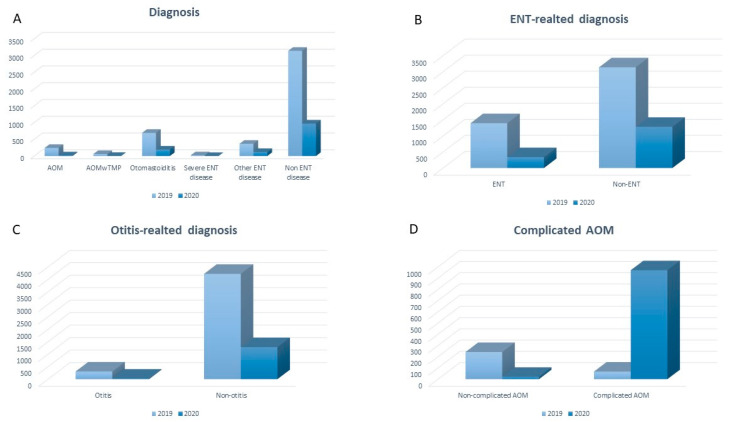
Distribution of all the diagnoses during the two study periods ((**A**): absolute numbers); comparison between the number of ENT- and non-ENT-related diagnoses (**B**); otitis- and non-otitis-related diagnoses (**C**), diagnoses of complicated acute otitis media episodes and diagnoses of non-complicated acute otitis media episodes (**D**) during the two study periods (ENT: ear, nose, throat; AOM: acute otitis media).

A total of 4538 children (mean age = 5.1 ± 5.3 years; males = 55.9%) were evaluated during period 1 compared to 1310 (mean age = 5.3 ± 6.6 years; males = 55.6%) during period 2 (*p* < 0.001). Populations were comparable in terms of main demographic characteristics (Table 2). The most frequent diagnoses were non-ENT-related ones (diagnostic group 7) in both periods, followed by non-complicated upper airway infections (diagnostic group 4); diagnoses related to middle ear infections (diagnostic groups 1–3) accounted for 6.8 and 1.8% of the diagnosis performed respectively in periods 1 and 2 (Table 2 and Figure 1).

A statistically significant overall effect on diagnosis between the study periods was attested (*p*-value < 0.001; pseudo R2 = 0.010) (Table 3) after adjustment for age and gender, the number of ENT-related diagnoses (diagnostic groups 1–6) being more frequently documented in period 1 compared to period 2 (*p*-value < 0.001), as well as those (diagnostic groups 1–3) related to all middle ear infections (respectively, during periods 1 and 2; *p*-value < 0.001) (Figure 1). Similarly, non-complicated AOM episodes without spontaneous tympanic membrane perforation (diagnostic group 1) were more frequently observed in period 1 compared to period 2 (*p*-value < 0.001); on the contrary, no significant difference in the number of complicated middle ear infections (diagnostic groups 2–3) between the two study periods was documented (*p*-value = non-significant) (Figure 1).

## 4. Discussion

Our study primarily focused on the impact of the Italian lockdown due to COVID-19 on the number of admissions for ENT-related disorders (including simple and complicated acute otitis media) at a large PED, placed in Milan’s city center, from March to May 2020.

As expected, we found a consistent global reduction in the number of children admitted to our PED during lockdown (period 2) compared to period 1. This is in line with the previous finding by Ibsa et al. [5], who compared the attendance of children/young patients at two large hospitals in Greater Manchester (UK) (i.e., a large district general hospital having a PED, and a regional children’s hospital) during February–March 2019 and February–March 2020. They found that patient attendance rates decreased by 5.6 and 30.4%, respectively, in the former and latter centers, with a larger reduction in March 2020 and an acceleration after the “UK lockdown” announcement on 23 March 2020.

Our results documented a statistically significant overall effect on diagnosis between the study periods as ENT-related diagnoses (diagnostic groups 1–6) were more frequently documented in period 1 compared to period 2, as well as those (diagnostic groups 1–3) more strictly related to middle ear infections. Similarly, non-complicated AOM episodes without spontaneous tympanic membrane perforation (diagnostic group 1) were more frequently observed during period 1 compared to period 2, while no significant difference in the number of diagnoses related to complicated middle ear infections (diagnostic groups 2–3) was documented.

Therefore, we found that the exceptional circumstances of the Italian lockdown resulted in a substantial regional decrease in children’s attendance to our PED as a whole; this effect was particularly conspicuous when analysis was restricted only to the ENT diagnoses, mainly non-complicated AOM (i.e., AOM without spontaneous tympanic membrane perforation or any other complication, including otomastoiditis). The finding of a significant time effect on diagnoses, with a remarkable effect especially on non-complicated or non-severe ENT diseases, is not simple to explain. However, one may consider that some healthcare policy measures aimed at discouraging both non-extremely urgent accesses to the PEDs and, more generally, any in-person visit to family pediatricians were introduced in our region at the outbreak of the pandemic. In addition, we think that the number of children presenting to healthcare facilities for minor clinical complaints such as the ones related to upper airway infections and simple AOM was affected also by the fear of being infected while going to hospital or outpatients clinics. These situations, in addition to limited access to pediatrician consultation or the use of telemedicine in the place of in-person visits, may have resulted, on the one hand, in an inadequate identification and treatment of simple middle ear infections, which subsequently evolved into complicated diseases, and, on the other hand, in selecting patients with complicated ENT disease who could be effectively managed only on a PED basis. This may explain the lack of a significant difference in the number of patients with complicated middle ear disease detected between periods in the present study.

A positive effect of the Italian lockdown on middle ear disease in children was confirmed by Aldè et al. [6], who documented a global reduction in the number of outpatient children with otitis media with effusion attending a pediatric audiological service in Milan during and after the lockdown, with a 93% resolution rate of chronic effusion in May–June 2020 compared to a 21% resolution rate noted in May–June 2019.

The impact of social distancing, school closure, and other lockdown strategies on clinical manifestations of middle ear and respiratory tract infections in children has been positively assessed by a few European papers [7,8]. In particular, Angoulvant et al. [7] in their time-series surveillance analysis based on data collected from a large multicenter French study attested to a global reduction in the number of both PED visits and hospital admissions after the lockdown declaration, with a >70% reduction in the incidence of some infectious diseases including AOM. Kuitunen et al. [8], studying the immediate effect of the Finnish national lockdown on PED visits and respiratory tract infections by means of a register-based study, found a major decrease in the daily rate of PED visits and an overall decrease in the global number of hospitalized patients.

Our results and literature analysis suggest that abolition of inter-personal contacts among children in school and day-care (initially ascribable to regionally restrictive measures, and then imposed by the national lockdown) resulted in fewer visits to our PED, especially for children receiving healthcare assistance for non-complicated AOM and non-severe ENT disease. This makes us suppose that the reduced number of viral infections among children occurring during the COVID-19 pandemic would have a positive impact on the natural history of middle ear infections not only in children with a chronic or recurrent form (i.e., otitis-prone children), as previously documented [2], but also in the general pediatric population living in Milan. In addition, the habit of wearing facial masks could reduce circulation of airborne disease and possibly influence the spreading of middle ear impairment triggered by airborne upper airway infections. The adoption of other hygienic measures such as frequent hand washing and the use of alcoholic hand disinfectant could partially account for this, too.

During the Italian lockdown, a reduction in air pollution was documented; this seemed to be particularly remarkable in the highly trafficked city of Milan [6,7], which generally has high atmospheric pollutant levels. Despite air pollution being significantly associated with the development of upper airway infections, the design of our study did not allow us to ascertain if the improvement in air quality documented in the city of Milan during the COVID-19 pandemic positively and independently contributed to the decrease in the middle ear complaints of children visiting our PED.

In any case, on the basis of our results, we can confirm the primary etiological role of some well-known risk factors for middle ear disease (day-care attendance, for one) that had long been considered unmodifiable predisposing conditions [8,9,10,11,12,13].

The main limitations of the present study are its retrospective design and the lack of comparison with data collected from other PEDs in our region\nation. In fact, the impact of lockdown strategies during the COVID-19 pandemic on middle ear infections in children could be different in geographic areas that were not as badly affected; this does not allow us to directly generalize the results of our city.

## 5. Conclusions

The exceptional circumstances of the Italian lockdown resulted in a significant general decrease in patients’ attendance at our PED in Milan’s city center. This effect was noted in most subgroups, but it was particularly prominent when considering diagnoses related to ENT disorders, middle ear diseases, and non-complicated middle ear infections.

However, our results are limited to our city and, given the lack of nationwide data, they cannot be generalized as they do not completely reflect the national and sovra-national situation during the pandemic.

Considering the not completely stable epidemiological situation here in Europe and the possibility of future national lockdowns, we ask ourselves what the months ahead will look like and how we can best prepare for them.

## Figures and Tables

**Table 1:** Diagnoses.

Macroarea	Diagnostic Group	ICD-9 Diagnosis	ICD-9 Code
Infectious middle ear disease	1	Non-complicated acute otitis media without spontaneous tympanic membrane perforation	[38200]
	2	Non-complicated acute otitis media with spontaneous tympanic membrane perforation	[38201]
	3	Complicated acute otitis media with otomastoiditis	[38300]
Non-complicated upper airway infections	4	Acute pharyngitis/tonsillitis	[462,463]
	4	Nasopharyngitis, common cold, and other upper airway infection	[460]
Other ENT diagnoses	5	Acute non-complicated rhinosinusitis	[4619]
	5	Suppurative lymphadenitis of the neck	[683]
	5	Acute laryngitis without airway obstruction	[46400]
	5	Epistaxis	[7847]
	5	Nasal trauma	[95909]
	5	Foreign bodies into the ear, nose, or oral cavity	[931,932,9350]
	5	External otitis	[38010]
Severe ENT disease	6	Periorbital cellulitis	[37601]
	6	Deep neck space abscess	[6821]
	6	Acute laryngitis with airway obstruction	[46401]
Non-ENT disease	7	Everything else	

Legend: ENT = ear nose and throat; ICD-9, International Classification of Disease-9.

**Table 2:** Demographic and clinical characteristics of the population.

Variable		Period 1 Tot. 4538	Period 2 Tot. 1310	*p*-Value	Global Tot. 5848
*N*. males (%)		2535 (55.9)	728 (55.6)	n.s.	3263 (55.8)
Mean age (sd), years		5.1 (5.3)	5.3 (6.6)	n.s.	5.1 (5.6)
Diagnosis	sAOM (%)	242 (5.3)	21 (1.6)	<0.001	263 (4.5)
	AOMwTMP (%)	59 (1.3)	3 (0.2)	n.s.	62 (1.1)
	AOMwM (%)	9 (0.2)	0 (0.0)	n.s.	9 (0.1)
	Non-complicated upper airway infections (%)	692 (15.3)	193 (14.7)	<0.001	885 (15.2)
	Other ENT diagnoses (%)	366 (8.1)	121 (9.3)	<0.001	487 (8.3)
	Severe ENT disease (%)	23 (0.5)	1 (0.1)	n.s.	24 (0.4)
	Non-ENT disease (%)	3147 (69.3)	971 (74.1)	<0.001	4118 (70.4)

Legend: *N*. = number; sd = standard deviation; n.s. = not significant; sAOM = simple acute otitis media; AOMwTMP = acute otitis media with spontaneous tympanic membrane perforation; AOMwM = acute otitis media with mastoiditis; ENT = ear nose and throat.

**Table 3:** Results of factorial logistic regression analysis testing the relationship between diagnosis and period.

*p*-Value = 0.0000				
Pseudo R2 = 0.0106				
Odds Ratio Standard Error *p*-value [95% Confidence Interval]				
Diagnosis 2	0.58	0.37	0.399	0.17–2.03
Diagnosis 4	3.21	0.78	0.000	2.00–5.16
Diagnosis 5	3.81	0.95	0.000	2.33–6.22
Diagnosis 6	0.50	0.52	0.509	0.06–3.90
Diagnosis 7	3.56	0.82	0.000	2.27–5.59

## Data Availability

Data are available from the corresponding author upon reasonable request.

## Abbreviations

PED	Pediatric Emergency Department
COVID	Coronavirus disease
n.	number
sd	standard deviation
sAOM	simple acute otitis media
AOMwTMP	acute otitis media with spontaneous tympanic membrane perforation
AOMwM	acute otitis media with mastoiditis
ENT	ear nose and throat
